# A new langbeinite-type phosphate: K_2_AlSn(PO_4_)_3_

**DOI:** 10.1107/S1600536811037263

**Published:** 2011-09-20

**Authors:** Hai-Yan Li, Dan Zhao

**Affiliations:** a Department of Physics and Chemistry, Henan Polytechnic University, Jiaozuo, Henan 454000, People’s Republic of China

## Abstract

Single crystals of the title compound, dipotassium aluminium tin(IV) tris­[phosphate(V)], K_2_AlSn(PO_4_)_3_, were synthesized by a high temperature reaction in a platinum crucible. In the structure, the Al^III^ and Sn^IV^ atoms occupy the same site on a threefold rotation axis with occupational disorder in a 1:1 ratio. In the three-dimensional structure, Al/SnO_6_ octa­hedra and PO_4_ tetra­hedra are inter­connected *via* their vertices, yielding a [Al/SnP_3_O_12_]_*n*_ framework. The K atoms (site symmetry 3) reside in the large cavities delimited by the [Al/SnP_3_O_12_]_*n*_ framework, and are surrounded by 12 O atoms.

## Related literature

For the mineral langbeinite, K_2_Mg_2_(SO_4_)_3_, see: Zemann & Zemann (1957 ▶). For related langbeinite-type compounds, see: Aatiq *et al.* (2006 ▶); Norberg (2002 ▶); Ogorodnyk *et al.* (2006 ▶); Orlova *et al.* (2003 ▶); Zatovsky *et al.* (2007 ▶); Zhao *et al.* (2009 ▶).

## Experimental

### 

#### Crystal data

K_2_AlSn(PO_4_)_3_
*M* *_r_* = 508.78
Cubic, 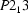

*a* = 9.7980 (8) Å
*V* = 940.62 (13) Å^3^
*Z* = 4
Mo *K*α radiation
μ = 4.28 mm^−1^
*T* = 296 K
0.15 × 0.05 × 0.05 mm

#### Data collection

Bruker APEXII CCD area-detector diffractometer
Absorption correction: multi-scan (*SADABS*; Sheldrick, 1996 ▶) *T*_min_ = 0.566, *T*_max_ = 0.815
6146 measured reflections
811 independent reflections
782 reflections with *I* > 2σ(*I*)
*R*_int_ = 0.065

#### Refinement

*R*[*F* ^2^ > 2σ(*F* ^2^)] = 0.029
*wR*(*F* ^2^) = 0.074
*S* = 1.18
811 reflections
59 parameters
Δρ_max_ = 0.53 e Å^−3^
Δρ_min_ = −0.60 e Å^−3^
Absolute structure: Flack (1983 ▶), 340 Friedel pairs
Flack parameter: −0.05 (7)

### 

Data collection: *APEX2* (Bruker, 2008 ▶); cell refinement: *SAINT* (Bruker, 2008 ▶); data reduction: *SAINT*; program(s) used to solve structure: *SHELXS97* (Sheldrick, 2008 ▶); program(s) used to refine structure: *SHELXL97* (Sheldrick, 2008 ▶); molecular graphics: *SHELXTL* (Sheldrick, 2008 ▶); software used to prepare material for publication: *SHELXTL*.

## Supplementary Material

Crystal structure: contains datablock(s) I, global. DOI: 10.1107/S1600536811037263/fi2112sup1.cif

Structure factors: contains datablock(s) I. DOI: 10.1107/S1600536811037263/fi2112Isup2.hkl

Additional supplementary materials:  crystallographic information; 3D view; checkCIF report

## Figures and Tables

**Fig. 1.**
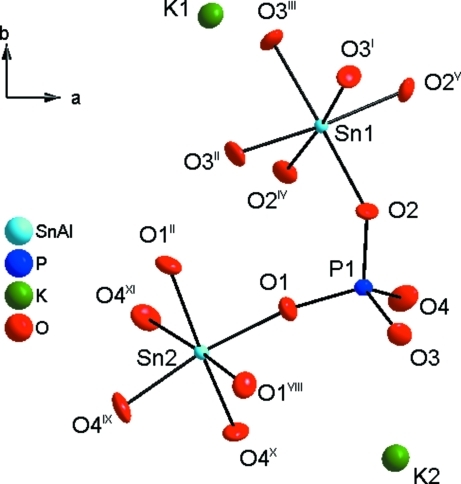
A view of the asymmetric unit of K2AlSn(PO4)3 showing the coordination environments of the P and Al/Sn atoms.. Displacement ellipsoids are drawn at the 50% probability level. [Symmetry codes: (i) -x + 1, y + 1/2, -z + 1/2; (ii) z, x, y; (iii) -y + 1/2, -z + 1, x - 1/2; (iv) -z + 1/2, -x + 1, y - 1/2; (v) -y + 1, z + 1/2, -x + 1/2; (viii) y, z, x; (ix) -z, x - 1/2, -y + 1/2; (x) -y + 1/2, -z, x - 1/2; (xi) x - 1/2, -y + 1/2, -z.]

**Fig. 2.**
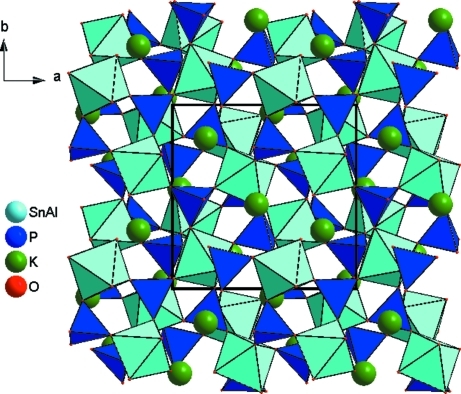
View of the crystal structure of K2AlSn(PO4)3 along [010]. PO4 and Al/SnO6 units are given in the polyhedral representation.

## supplementary crystallographic information

### Comment

Langbeinite-type (K_2_Mg_2_(SO_4_)_3_, Zemann & Zemann, 1957) compounds with the simplest generic formula *A*_2_*B*_2_(*X*O_4_)_3_ are an important and well studied family of inorganic solids with respect to minerals. Among Langbeinite-based phosphates, whose coordination networks are based on [*M*_2_(PO_4_)_3_] fragments, may result in diverse structure types due to the '*M*_2_' sites occupied by various types of tetravalent and bi- or trivalent metal pairs. For example, the structures K_2_*M*Ti(PO_4_)_3_(*M* = Y, Yb, Er) (Norberg, 2002), K_2_FeZr(PO_4_)_3_ (Orlova *et al.*, 2003), K_2_*M*Sn(PO_4_)_3_ (*M* = Fe, Yb) (Aatiq *et al.*, 2006), K_2_AlTi(PO_4_)_3_ (Zhao *et al.*, 2009), K_2_FeSn(PO_4_)_3_ (Zatovsky *et al.*, 2007) and K_2_Mn_0.5_Ti_1.5_(PO_4_)_3_ (Ogorodnyk *et al.*, 2006), have been reported. Herein we report the single-crystal growth and structure investigation of title compound K_2_AlSn(PO_4_)_3_.

In the structure of title compound, K, Al and Sn atoms lie on the 3-fold rotation axes in 4a positions, while P and O atoms are located at general 12*b* positions. Due to the similar ionic radii of Al and Sn atoms, they occupy the same sites in a substituent disordered manner, denoted as *M* atoms. The three-dimensional structure contains *M*O_6_ octahedra and PO_4_ tetrahedra which are connected *via* vertices. Two nearest [*M*O_6_] octahedra are joined to each other by three bridging orthophosphate tetrahedra forming [Al/SnP_3_O_12_]_n_ framework, which penetrate with large closed cavities. Two independent potassium atoms are located in each cavity. K1 and K2 atoms are 12-coordinated by O atoms.

### Experimental

Single crystals of K_2_AlSn(PO_4_)_3_ have been prepared by a high-temperature method in air. A powder mixture of K_2_CO_3_, Al_2_O_3_, SnO_2_ and NH_4_H_2_PO_4_ in the molar ratio of K: Al: Sn: P = 10: 1:: 1 15 was first ground in an agate mortar and then transferred to a platinum crucible. The sample was gradually heated in air at 1173 K for 24 h. After that, the intermediate product was slowly cooled to 673 K at the rate of 2 K h^-1^. It was kept at 673 K for another 10 h and then quenched to room temperature. The obtained crystals were colorless with a prismatic shape.

### Refinement

The atomic position and anisotropic displacement parameters of Al and Sn in the same sites were constrained to be identical, and the Al/Sn disorder with a relative occupancy of 1/1. The highest peak in the difference electron density map equals to 0.53 e/Å^3^ at the distance of 1.09 Å from Al2/Sn2 site while the deepest hole equals to -0.60 e/Å^3^ at the distance of 1.78 Å from K2 site.

### Crystal data

**Table:**

K_2_AlSn(PO_4_)_3_	*D*_x_ = 3.593 Mg m^−^^3^
*M**_r_* = 508.78	Mo *K*α radiation, λ = 0.71073 Å
Cubic, *P*2_1_3	Cell parameters from 2030 reflections
Hall symbol: P 2ac 2ab 3	θ = 3.6–28.5°
*a* = 9.7980 (8) Å	µ = 4.28 mm^−^^1^
*V* = 940.62 (13) Å^3^	*T* = 296 K
*Z* = 4	Prism, colourless
*F*(000) = 968	0.15 × 0.05 × 0.05 mm

### Data collection

**Table:**

Bruker SMART APEXII CCD area-detector diffractometer	811 independent reflections
Radiation source: fine-focus sealed tube	782 reflections with *I* > 2σ(*I*)
graphite	*R*_int_ = 0.065
Detector resolution: 83.33 pixels mm^-1^	θ_max_ = 28.5°, θ_min_ = 2.9°
ω scans	*h* = −13→6
Absorption correction: multi-scan (*SADABS*; Sheldrick, 1996)	*k* = −12→12
*T*_min_ = 0.566, *T*_max_ = 0.815	*l* = −12→10
6146 measured reflections	

### Refinement

**Table:**

Refinement on *F*^2^	Secondary atom site location: difference Fourier map
Least-squares matrix: full	*w* = 1/[σ^2^(*F*_o_^2^) + (0.0208*P*)^2^ + 3.2535*P*] where *P* = (*F*_o_^2^ + 2*F*_c_^2^)/3
*R*[*F*^2^ > 2σ(*F*^2^)] = 0.029	(Δ/σ)_max_ < 0.001
*wR*(*F*^2^) = 0.074	Δρ_max_ = 0.53 e Å^−^^3^
*S* = 1.18	Δρ_min_ = −0.60 e Å^−^^3^
811 reflections	Extinction correction: *SHELXTL* (Sheldrick, 2008), Fc^*^=kFc[1+0.001xFc^2^λ^3^/sin(2θ)]^-1/4^
59 parameters	Extinction coefficient: 0.0076 (10)
0 restraints	Absolute structure: Flack (1983), 340 Friedel pairs
Primary atom site location: structure-invariant direct methods	Flack parameter: −0.05 (7)

### Special details

**Table:**

Geometry. All e.s.d.'s (except the e.s.d. in the dihedral angle between two l.s. planes) are estimated using the full covariance matrix. The cell e.s.d.'s are taken into account individually in the estimation of e.s.d.'s in distances, angles and torsion angles; correlations between e.s.d.'s in cell parameters are only used when they are defined by crystal symmetry. An approximate (isotropic) treatment of cell e.s.d.'s is used for estimating e.s.d.'s involving l.s. planes.
Refinement. Refinement of *F*^2^ against ALL reflections. The weighted *R*-factor *wR* and goodness of fit *S* are based on *F*^2^, conventional *R*-factors *R* are based on *F*, with *F* set to zero for negative *F*^2^. The threshold expression of *F*^2^ > σ(*F*^2^) is used only for calculating *R*-factors(gt) *etc*. and is not relevant to the choice of reflections for refinement. *R*-factors based on *F*^2^ are statistically about twice as large as those based on *F*, and *R*- factors based on ALL data will be even larger.

### Fractional atomic coordinates and isotropic or equivalent isotropic displacement parameters (Å^2^)

**Table:**

	*x*	*y*	*z*	*U*_iso_*/*U*_eq_	Occ. (<1)
Sn1	0.39682 (6)	0.60318 (6)	0.10318 (6)	0.0091 (2)	0.50
Sn2	0.16412 (6)	0.16412 (6)	0.16412 (6)	0.0104 (2)	0.50
Al1	0.39682 (6)	0.60318 (6)	0.10318 (6)	0.0091 (2)	0.50
Al2	0.16412 (6)	0.16412 (6)	0.16412 (6)	0.0104 (2)	0.50
P1	0.47928 (15)	0.29006 (15)	0.12495 (15)	0.0133 (3)	
K1	0.18221 (14)	0.81779 (14)	0.31779 (14)	0.0268 (5)	
K2	0.54175 (17)	−0.04175 (17)	0.04175 (17)	0.0305 (6)	
O1	0.3329 (4)	0.2484 (4)	0.1004 (5)	0.0231 (9)	
O2	0.4861 (4)	0.4378 (4)	0.1734 (5)	0.0198 (9)	
O3	0.5491 (5)	0.1991 (4)	0.2297 (4)	0.0214 (9)	
O4	0.5569 (5)	0.2684 (5)	−0.0094 (5)	0.0276 (11)	

### Atomic displacement parameters (Å^2^)

**Table:**

	*U*^11^	*U*^22^	*U*^33^	*U*^12^	*U*^13^	*U*^23^
Sn1	0.0091 (2)	0.0091 (2)	0.0091 (2)	0.0000 (2)	0.0000 (2)	0.0000 (2)
Sn2	0.0104 (2)	0.0104 (2)	0.0104 (2)	−0.0010 (2)	−0.0010 (2)	−0.0010 (2)
Al1	0.0091 (2)	0.0091 (2)	0.0091 (2)	0.0000 (2)	0.0000 (2)	0.0000 (2)
Al2	0.0104 (2)	0.0104 (2)	0.0104 (2)	−0.0010 (2)	−0.0010 (2)	−0.0010 (2)
P1	0.0142 (7)	0.0131 (7)	0.0126 (7)	0.0002 (5)	−0.0001 (5)	0.0010 (5)
K1	0.0268 (5)	0.0268 (5)	0.0268 (5)	0.0031 (6)	0.0031 (6)	−0.0031 (6)
K2	0.0305 (6)	0.0305 (6)	0.0305 (6)	0.0013 (6)	−0.0013 (6)	0.0013 (6)
O1	0.016 (2)	0.024 (2)	0.030 (2)	−0.0048 (18)	−0.006 (2)	0.0024 (19)
O2	0.021 (2)	0.014 (2)	0.025 (2)	−0.0009 (16)	−0.0065 (19)	−0.0067 (17)
O3	0.025 (2)	0.021 (2)	0.018 (2)	−0.0001 (18)	−0.0077 (18)	0.0088 (18)
O4	0.038 (3)	0.028 (3)	0.017 (2)	0.007 (2)	0.011 (2)	0.003 (2)

### Geometric parameters (Å, °)

**Table:**

Sn1—O3^i^	1.961 (4)	K1—O4^xii^	3.011 (6)
Sn1—O3^ii^	1.961 (4)	K1—O4^xi^	3.011 (6)
Sn1—O3^iii^	1.961 (4)	K1—O4^ii^	3.208 (5)
Sn1—O2^iv^	1.966 (4)	K1—O4^i^	3.208 (5)
Sn1—O2^v^	1.966 (4)	K1—O4^iii^	3.208 (5)
Sn1—O2	1.966 (4)	K2—O2^xiii^	2.811 (5)
Sn2—O1	1.951 (4)	K2—O2^xiv^	2.811 (5)
Sn2—O1^ii^	1.951 (4)	K2—O2^xv^	2.811 (5)
Sn2—O1^vi^	1.951 (4)	K2—O3^ix^	2.994 (5)
Sn2—O4^vii^	1.960 (5)	K2—O3^xvi^	2.994 (5)
Sn2—O4^viii^	1.960 (5)	K2—O3	2.994 (5)
Sn2—O4^ix^	1.960 (5)	K2—O4^xvi^	3.084 (5)
P1—O1	1.511 (4)	K2—O4^ix^	3.084 (5)
P1—O2	1.525 (4)	K2—O4	3.084 (5)
P1—O3	1.521 (4)	O1—K1^xvii^	2.847 (5)
P1—O4	1.535 (5)	O2—K2^xviii^	2.811 (5)
K1—O1^x^	2.847 (5)	O3—Al1^vi^	1.961 (4)
K1—O1^xi^	2.847 (5)	O3—Sn1^vi^	1.961 (4)
K1—O1^xii^	2.847 (5)	O3—K1^vi^	2.915 (5)
K1—O3^ii^	2.916 (5)	O4—Al2^xvi^	1.960 (5)
K1—O3^iii^	2.916 (5)	O4—Sn2^xvi^	1.960 (5)
K1—O3^i^	2.916 (5)	O4—K1^xvii^	3.011 (6)
K1—O4^x^	3.011 (6)	O4—K1^vi^	3.208 (5)
			
O3^i^—Sn1—O3^ii^	87.42 (19)	O4^x^—K1—O4^xii^	86.48 (14)
O3^i^—Sn1—O3^iii^	87.42 (19)	O1^x^—K1—O4^xi^	52.14 (13)
O3^ii^—Sn1—O3^iii^	87.42 (19)	O1^xi^—K1—O4^xi^	49.38 (13)
O3^i^—Sn1—O2^iv^	175.0 (2)	O1^xii^—K1—O4^xi^	114.88 (15)
O3^ii^—Sn1—O2^iv^	89.00 (17)	O3^ii^—K1—O4^xi^	132.86 (13)
O3^iii^—Sn1—O2^iv^	88.90 (18)	O3^iii^—K1—O4^xi^	95.45 (12)
O3^i^—Sn1—O2^v^	88.90 (19)	O3^i^—K1—O4^xi^	140.66 (13)
O3^ii^—Sn1—O2^v^	175.0 (2)	O4^x^—K1—O4^xi^	86.48 (14)
O3^iii^—Sn1—O2^v^	89.00 (17)	O4^xii^—K1—O4^xi^	86.48 (14)
O2^iv^—Sn1—O2^v^	94.46 (18)	O1^x^—K1—O4^ii^	156.33 (13)
O3^i^—Sn1—O2	88.99 (17)	O1^xi^—K1—O4^ii^	55.82 (12)
O3^ii^—Sn1—O2	88.90 (18)	O1^xii^—K1—O4^ii^	86.26 (12)
O3^iii^—Sn1—O2	175.0 (2)	O3^ii^—K1—O4^ii^	46.65 (11)
O2^iv^—Sn1—O2	94.46 (18)	O3^iii^—K1—O4^ii^	88.29 (13)
O2^v^—Sn1—O2	94.45 (18)	O3^i^—K1—O4^ii^	100.74 (13)
O3^i^—Sn1—K1	52.93 (13)	O4^x^—K1—O4^ii^	136.85 (10)
O3^ii^—Sn1—K1	52.93 (13)	O4^xii^—K1—O4^ii^	53.57 (17)
O3^iii^—Sn1—K1	52.93 (13)	O4^xi^—K1—O4^ii^	104.39 (2)
O2^iv^—Sn1—K1	122.05 (13)	O1^x^—K1—O4^i^	86.26 (12)
O2^v^—Sn1—K1	122.05 (13)	O1^xi^—K1—O4^i^	156.33 (13)
O2—Sn1—K1	122.05 (13)	O1^xii^—K1—O4^i^	55.82 (12)
O3^i^—Sn1—K2^ii^	129.38 (14)	O3^ii^—K1—O4^i^	88.29 (13)
O3^ii^—Sn1—K2^ii^	51.14 (14)	O3^iii^—K1—O4^i^	100.74 (13)
O3^iii^—Sn1—K2^ii^	66.00 (13)	O3^i^—K1—O4^i^	46.65 (11)
O2^iv^—Sn1—K2^ii^	45.73 (13)	O4^x^—K1—O4^i^	53.57 (17)
O2^v^—Sn1—K2^ii^	130.02 (13)	O4^xii^—K1—O4^i^	104.39 (3)
O2—Sn1—K2^ii^	114.01 (13)	O4^xi^—K1—O4^i^	136.85 (10)
K1—Sn1—K2^ii^	77.229 (18)	O4^ii^—K1—O4^i^	115.74 (6)
O3^i^—Sn1—K2^xix^	66.00 (13)	O1^x^—K1—O4^iii^	55.82 (12)
O3^ii^—Sn1—K2^xix^	129.38 (14)	O1^xi^—K1—O4^iii^	86.26 (12)
O3^iii^—Sn1—K2^xix^	51.14 (14)	O1^xii^—K1—O4^iii^	156.33 (13)
O2^iv^—Sn1—K2^xix^	114.01 (13)	O3^ii^—K1—O4^iii^	100.74 (13)
O2^v^—Sn1—K2^xix^	45.73 (13)	O3^iii^—K1—O4^iii^	46.65 (11)
O2—Sn1—K2^xix^	130.02 (13)	O3^i^—K1—O4^iii^	88.29 (13)
K1—Sn1—K2^xix^	77.229 (18)	O4^x^—K1—O4^iii^	104.39 (2)
K2^ii^—Sn1—K2^xix^	115.259 (13)	O4^xii^—K1—O4^iii^	136.85 (10)
O3^i^—Sn1—K2^xviii^	51.14 (14)	O4^xi^—K1—O4^iii^	53.57 (17)
O3^ii^—Sn1—K2^xviii^	66.00 (13)	O4^ii^—K1—O4^iii^	115.74 (6)
O3^iii^—Sn1—K2^xviii^	129.38 (14)	O4^i^—K1—O4^iii^	115.74 (6)
O2^iv^—Sn1—K2^xviii^	130.02 (13)	O2^xiii^—K2—O2^xiv^	91.85 (14)
O2^v^—Sn1—K2^xviii^	114.01 (13)	O2^xiii^—K2—O2^xv^	91.85 (14)
O2—Sn1—K2^xviii^	45.73 (13)	O2^xiv^—K2—O2^xv^	91.85 (14)
K1—Sn1—K2^xviii^	77.229 (18)	O2^xiii^—K2—O3^ix^	147.18 (15)
K2^ii^—Sn1—K2^xviii^	115.259 (13)	O2^xiv^—K2—O3^ix^	56.50 (11)
K2^xix^—Sn1—K2^xviii^	115.259 (13)	O2^xv^—K2—O3^ix^	81.71 (12)
O1—Sn2—O1^ii^	92.8 (2)	O2^xiii^—K2—O3^xvi^	56.50 (11)
O1—Sn2—O1^vi^	92.8 (2)	O2^xiv^—K2—O3^xvi^	81.71 (12)
O1^ii^—Sn2—O1^vi^	92.8 (2)	O2^xv^—K2—O3^xvi^	147.18 (15)
O1—Sn2—O4^vii^	93.7 (2)	O3^ix^—K2—O3^xvi^	119.27 (3)
O1^ii^—Sn2—O4^vii^	82.5 (2)	O2^xiii^—K2—O3	81.71 (12)
O1^vi^—Sn2—O4^vii^	172.2 (2)	O2^xiv^—K2—O3	147.18 (15)
O1—Sn2—O4^viii^	172.2 (2)	O2^xv^—K2—O3	56.50 (11)
O1^ii^—Sn2—O4^viii^	93.7 (2)	O3^ix^—K2—O3	119.27 (3)
O1^vi^—Sn2—O4^viii^	82.5 (2)	O3^xvi^—K2—O3	119.27 (3)
O4^vii^—Sn2—O4^viii^	91.5 (2)	O2^xiii^—K2—O4^xvi^	103.66 (12)
O1—Sn2—O4^ix^	82.5 (2)	O2^xiv^—K2—O4^xvi^	82.44 (13)
O1^ii^—Sn2—O4^ix^	172.2 (2)	O2^xv^—K2—O4^xvi^	163.58 (13)
O1^vi^—Sn2—O4^ix^	93.7 (2)	O3^ix^—K2—O4^xvi^	82.31 (13)
O4^vii^—Sn2—O4^ix^	91.5 (2)	O3^xvi^—K2—O4^xvi^	47.30 (12)
O4^viii^—Sn2—O4^ix^	91.5 (2)	O3—K2—O4^xvi^	130.38 (15)
O1—Sn2—K1^xx^	128.15 (13)	O2^xiii^—K2—O4^ix^	163.58 (13)
O1^ii^—Sn2—K1^xx^	49.01 (14)	O2^xiv^—K2—O4^ix^	103.66 (12)
O1^vi^—Sn2—K1^xx^	118.40 (13)	O2^xv^—K2—O4^ix^	82.44 (13)
O4^vii^—Sn2—K1^xx^	53.89 (16)	O3^ix^—K2—O4^ix^	47.30 (12)
O4^viii^—Sn2—K1^xx^	59.65 (16)	O3^xvi^—K2—O4^ix^	130.38 (15)
O4^ix^—Sn2—K1^xx^	130.31 (15)	O3—K2—O4^ix^	82.31 (13)
O1—Sn2—K1^xxi^	118.40 (13)	O4^xvi^—K2—O4^ix^	83.98 (16)
O1^ii^—Sn2—K1^xxi^	128.15 (13)	O2^xiii^—K2—O4	82.44 (13)
O1^vi^—Sn2—K1^xxi^	49.01 (14)	O2^xiv^—K2—O4	163.58 (13)
O4^vii^—Sn2—K1^xxi^	130.31 (15)	O2^xv^—K2—O4	103.66 (12)
O4^viii^—Sn2—K1^xxi^	53.89 (16)	O3^ix^—K2—O4	130.38 (15)
O4^ix^—Sn2—K1^xxi^	59.65 (16)	O3^xvi^—K2—O4	82.31 (13)
K1^xx^—Sn2—K1^xxi^	113.210 (15)	O3—K2—O4	47.30 (12)
O1—Sn2—K1^xvii^	49.01 (14)	O4^xvi^—K2—O4	83.98 (16)
O1^ii^—Sn2—K1^xvii^	118.40 (13)	O4^ix^—K2—O4	83.98 (16)
O1^vi^—Sn2—K1^xvii^	128.15 (13)	O2^xiii^—K2—P1	93.94 (9)
O4^vii^—Sn2—K1^xvii^	59.65 (16)	O2^xiv^—K2—P1	169.50 (10)
O4^viii^—Sn2—K1^xvii^	130.31 (15)	O2^xv^—K2—P1	79.23 (9)
O4^ix^—Sn2—K1^xvii^	53.89 (16)	O3^ix^—K2—P1	116.07 (10)
K1^xx^—Sn2—K1^xvii^	113.210 (15)	O3^xvi^—K2—P1	108.78 (10)
K1^xxi^—Sn2—K1^xvii^	113.210 (15)	O3—K2—P1	26.50 (8)
O1—P1—O2	110.3 (3)	O4^xvi^—K2—P1	104.63 (13)
O1—P1—O3	112.1 (3)	O4^ix^—K2—P1	69.91 (10)
O2—P1—O3	109.1 (3)	O4—K2—P1	26.76 (9)
O1—P1—O4	107.2 (3)	O2^xiii^—K2—P1^ix^	169.50 (10)
O2—P1—O4	112.1 (3)	O2^xiv^—K2—P1^ix^	79.23 (9)
O3—P1—O4	105.9 (3)	O2^xv^—K2—P1^ix^	93.94 (9)
O1—P1—K1^vi^	168.93 (19)	O3^ix^—K2—P1^ix^	26.50 (8)
O2—P1—K1^vi^	80.16 (17)	O3^xvi^—K2—P1^ix^	116.07 (10)
O3—P1—K1^vi^	59.55 (18)	O3—K2—P1^ix^	108.78 (10)
O4—P1—K1^vi^	70.5 (2)	O4^xvi^—K2—P1^ix^	69.91 (10)
O1—P1—K2	82.78 (18)	O4^ix^—K2—P1^ix^	26.76 (9)
O2—P1—K2	166.53 (19)	O4—K2—P1^ix^	104.63 (13)
O3—P1—K2	61.42 (18)	P1—K2—P1^ix^	95.73 (6)
O4—P1—K2	64.79 (19)	O2^xiii^—K2—P1^xvi^	79.23 (9)
K1^vi^—P1—K2	86.56 (5)	O2^xiv^—K2—P1^xvi^	93.94 (9)
O1—P1—K1^xvii^	50.43 (19)	O2^xv^—K2—P1^xvi^	169.50 (10)
O2—P1—K1^xvii^	124.36 (19)	O3^ix^—K2—P1^xvi^	108.78 (10)
O3—P1—K1^xvii^	126.57 (19)	O3^xvi^—K2—P1^xvi^	26.50 (8)
O4—P1—K1^xvii^	56.9 (2)	O3—K2—P1^xvi^	116.07 (10)
K1^vi^—P1—K1^xvii^	126.95 (5)	O4^xvi^—K2—P1^xvi^	26.76 (9)
K2—P1—K1^xvii^	66.07 (6)	O4^ix^—K2—P1^xvi^	104.63 (13)
O1—P1—K2^xviii^	102.1 (2)	O4—K2—P1^xvi^	69.91 (10)
O2—P1—K2^xviii^	45.40 (18)	P1—K2—P1^xvi^	95.73 (6)
O3—P1—K2^xviii^	71.80 (18)	P1^ix^—K2—P1^xvi^	95.73 (6)
O4—P1—K2^xviii^	148.7 (2)	P1—O1—Sn2	150.2 (3)
K1^vi^—P1—K2^xviii^	82.58 (4)	P1—O1—K1^xvii^	105.4 (2)
K2—P1—K2^xviii^	130.74 (6)	Sn2—O1—K1^xvii^	99.85 (17)
K1^xvii^—P1—K2^xviii^	149.53 (5)	P1—O2—Sn1	130.9 (3)
O1^x^—K1—O1^xi^	100.53 (12)	P1—O2—K2^xviii^	111.9 (2)
O1^x^—K1—O1^xii^	100.53 (12)	Sn1—O2—K2^xviii^	104.22 (16)
O1^xi^—K1—O1^xii^	100.53 (12)	P1—O3—Al1^vi^	164.9 (3)
O1^x^—K1—O3^ii^	149.59 (14)	P1—O3—Sn1^vi^	164.9 (3)
O1^xi^—K1—O3^ii^	96.38 (13)	Al1^vi^—O3—Sn1^vi^	0.00 (5)
O1^xii^—K1—O3^ii^	100.99 (13)	P1—O3—K1^vi^	93.7 (2)
O1^x^—K1—O3^iii^	96.38 (13)	Al1^vi^—O3—K1^vi^	94.60 (17)
O1^xi^—K1—O3^iii^	100.99 (13)	Sn1^vi^—O3—K1^vi^	94.60 (17)
O1^xii^—K1—O3^iii^	149.59 (14)	P1—O3—K2	92.1 (2)
O3^ii^—K1—O3^iii^	55.40 (14)	Al1^vi^—O3—K2	98.18 (17)
O1^x^—K1—O3^i^	100.99 (13)	Sn1^vi^—O3—K2	98.18 (17)
O1^xi^—K1—O3^i^	149.59 (14)	K1^vi^—O3—K2	103.77 (14)
O1^xii^—K1—O3^i^	96.38 (13)	P1—O4—Al2^xvi^	152.0 (3)
O3^ii^—K1—O3^i^	55.40 (14)	P1—O4—Sn2^xvi^	152.0 (3)
O3^iii^—K1—O3^i^	55.40 (14)	Al2^xvi^—O4—Sn2^xvi^	0.000 (18)
O1^x^—K1—O4^x^	49.38 (13)	P1—O4—K1^xvii^	97.8 (2)
O1^xi^—K1—O4^x^	114.88 (15)	Al2^xvi^—O4—K1^xvii^	94.39 (18)
O1^xii^—K1—O4^x^	52.14 (13)	Sn2^xvi^—O4—K1^xvii^	94.39 (18)
O3^ii^—K1—O4^x^	140.66 (13)	P1—O4—K2	88.5 (2)
O3^iii^—K1—O4^x^	132.86 (13)	Al2^xvi^—O4—K2	118.9 (2)
O3^i^—K1—O4^x^	95.45 (12)	Sn2^xvi^—O4—K2	118.9 (2)
O1^x^—K1—O4^xii^	114.88 (15)	K1^xvii^—O4—K2	77.14 (14)
O1^xi^—K1—O4^xii^	52.14 (13)	P1—O4—K1^vi^	82.7 (2)
O1^xii^—K1—O4^xii^	49.38 (13)	Al2^xvi^—O4—K1^vi^	88.54 (19)
O3^ii^—K1—O4^xii^	95.45 (12)	Sn2^xvi^—O4—K1^vi^	88.54 (19)
O3^iii^—K1—O4^xii^	140.66 (13)	K1^xvii^—O4—K1^vi^	172.37 (18)
O3^i^—K1—O4^xii^	132.86 (12)	K2—O4—K1^vi^	95.27 (14)

Symmetry codes: (i) −*x*+1, *y*+1/2, −*z*+1/2; (ii) *z*, *x*, *y*; (iii) −*y*+1/2, −*z*+1, *x*−1/2; (iv) −*z*+1/2, −*x*+1, *y*−1/2; (v) −*y*+1, *z*+1/2, −*x*+1/2; (vi) *y*, *z*, *x*; (vii) *x*−1/2, −*y*+1/2, −*z*; (viii) −*z*, *x*−1/2, −*y*+1/2; (ix) −*y*+1/2, −*z*, *x*−1/2; (x) *y*, *z*+1, *x*; (xi) −*z*, *x*+1/2, −*y*+1/2; (xii) −*x*+1/2, −*y*+1, *z*+1/2; (xiii) −*z*+1, *x*−1/2, −*y*+1/2; (xiv) −*y*+1, *z*−1/2, −*x*+1/2; (xv) −*x*+1, *y*−1/2, −*z*+1/2; (xvi) *z*+1/2, −*x*+1/2, −*y*; (xvii) *z*, *x*, *y*−1; (xviii) −*z*+1/2, −*x*+1, *y*+1/2; (xix) *x*, *y*+1, *z*; (xx) *y*−1, *z*, *x*; (xxi) *x*, *y*−1, *z*.
